# Thermal conductivity analysis and applications of nanocellulose materials

**DOI:** 10.1080/14686996.2017.1390692

**Published:** 2017-11-03

**Authors:** Kojiro Uetani, Kimihito Hatori

**Affiliations:** ^a^ Department of Chemistry, College of Science, Rikkyo University, Tokyo, Japan; ^b^ Hudson Laboratory, Bethel Co., Ltd., Ibaraki, Japan

**Keywords:** thermal conductivity, nanocellulose, polymer, paper, composites, 20 Organic and soft materials (Colloids, liquid crystals, gels, polymers), 102 Porous / Nanoporous / Nanostructured materials, 210 Thermoelectronics / Thermal transport / insulators

## Abstract

In this review, we summarize the recent progress in thermal conductivity analysis of nanocellulose materials called cellulose nanopapers, and compare them with polymeric materials, including neat polymers, composites, and traditional paper. It is important to individually measure the in-plane and through-plane heat-conducting properties of two-dimensional planar materials, so steady-state and non-equilibrium methods, in particular the laser spot periodic heating radiation thermometry method, are reviewed. The structural dependency of cellulose nanopaper on thermal conduction is described in terms of the crystallite size effect, fibre orientation, and interfacial thermal resistance between fibres and small pores. The novel applications of cellulose as thermally conductive transparent materials and thermal-guiding materials are also discussed.

## Graphical abstract

**Figure:**
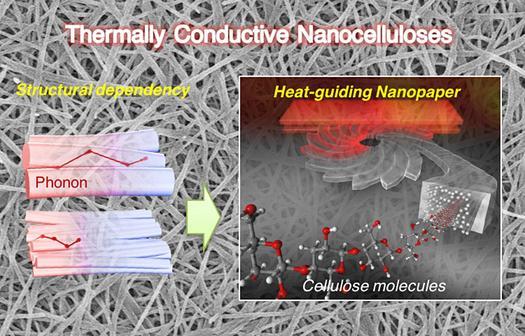

## 1. Introduction

Cellulose is the most ubiquitous biopolymer. It is synthesized by various living organisms, such as plants, to construct their tissues with unique hierarchical structures, including microfibrils, fibril bundles, and cell walls. The tissue structures have been widely used in human daily life as papers and clothes for several thousand years. Recently, cellulose microfibrils have attracted a great deal of attention from industrial fields owing to their excellent physical properties that sustain externally forced plant bodies. To fulfil the potential of cellulose microfibrils as future industrial materials, it is necessary to disintegrate the fine cell wall structures to obtain individual microfibrils or nanofibrous forms, so-called nanocelluloses (NCs). The individualization protocols to prepare NCs vary the NC morphologies (thickness, length, and curvature) and lead to a variety of mechanical, optical, and thermal functions.

**Figure 1.**
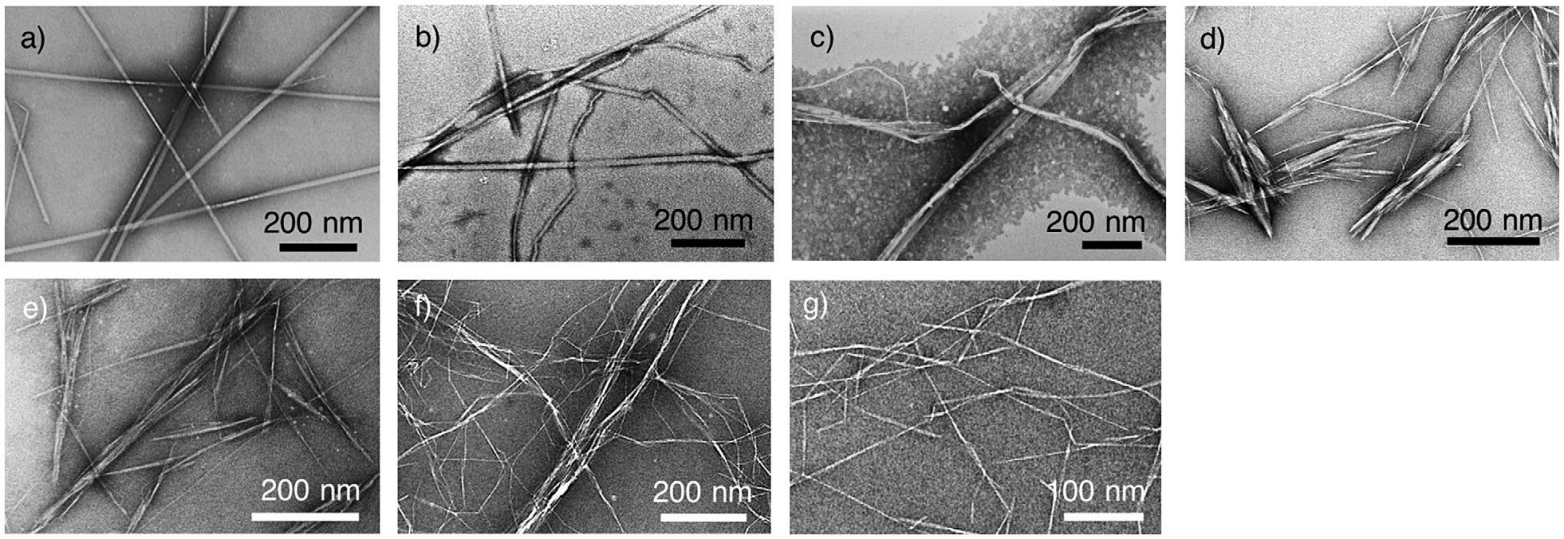
Transmission electron microscopy images of various NC materials. (a) Tunicate nanowhiskers, (b) tunicate nanofibres, (c) BC nanofibres, (d) cotton nanowhiskers, (e) Japanese cedar nanowhiskers, (f) Japanese cedar nanofibres, and (g) 2,2,6,6-tetramethylpiperidine-1-oxyl (TEMPO) radical-oxidized nanofibres of Japanese cedar cellulose. Reprinted with permission from [73]. Copyright 2015 American Chemical Society.

NCs include relatively long curved cellulose nanofibres (CNFs) and relatively short rod-like cellulose nanowhiskers (CNWs), as shown in Figure 1. CNFs are generally produced by chemical [1–3] and physical [4–8] nanofibrillation techniques. The hierarchical tissue architectures of living organisms, such as wood [1,3–5,8,9], kenaf [9], potato tuber [10], rice straw [9,10] wheat straw [6,11], bamboo [6], algae [12], and tunicate [13,14], are disintegrated to obtain CNFs. CNWs are produced by acid hydrolysis [15,16] of cellulosic resources, including wood [17,18], cotton [19,20], ramie [21], hemp [22], flax [23], sisal [24], rice straw [25], bacterial cellulose (BC) [26,27], and tunicate [20,28–30], to extract only the crystalline region of cellulose [31]. These NC materials consist of the long crystalline structure of cellulose [32], in which glucose rings linked with *β*-1,4 glycosidic linkage are linearly repeated. The crystallites of cellulose I type are the structural origin of the unique properties of NCs, such as the high Young’s modulus of 140–150 GPa [33,34], the high mechanical strength of 2–6 GPa [35], and the low coefficient of thermal expansion (CTE) of 6 ppm/K [36].

**Figure 2.**
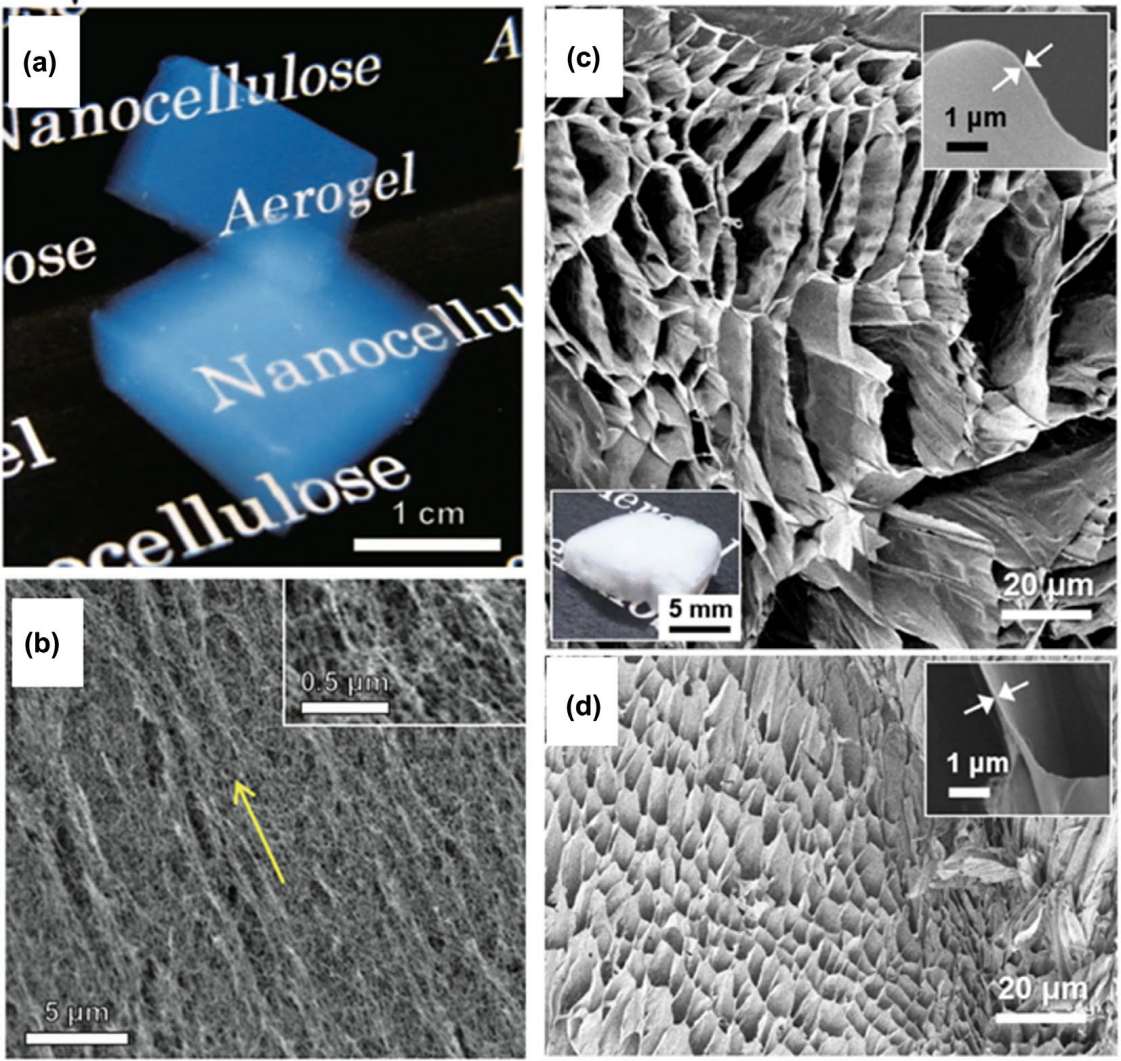
Porous NC materials. (a) Liquid-crystalline NC aerogel prepared by supercritical drying and (b) its scanning electron micrograph. Reprinted with permission from [47]. Copyright 2014 John Wiley and Sons. (c) and (d) Ultrahigh porosity foams with solid volume fractions of 0.32% and 1.04%, respectively. Reprinted with permission from [48]. Copyright 2016 Nature Publishing Group.

One of the main applications of cellulose is thermal insulation. Thermal insulation paper for electrical transformers [37,38] and loosely filled building insulating materials [39–44] can be produced from cellulosic fibres, including cotton stalk fibres [42], waste paper [43], and flax and hemp fibres [44]. Recently, NCs have also been used to produce low-density foams [45] and aerogels [46–49] (Figure 2), and composites with chitosan [50], polymethylsilsesquioxane [51], zeolites [52], silica [53], and graphene oxide [54] that have characteristic low thermal conductivities (i.e. high thermal insulation capacities). The thermal conductivities of general building insulators are below 0.1 W/mK. Some high-performance aerogels [47,48,54] have lower thermal conductivities than atmospheric air, which has a thermal conductivity of 0.0262 W/mK at 300 K under 0.1 MPa [55]. Despite the variety of thermal management applications, knowledge about the heat-conducting properties of cellulose has been limited until quite recently. Some studies have reported enhancement of the thermal conductivity of polymer matrices by mixing them with NCs [56,57], indicating that NCs might have high thermal conductivity.

**Figure 3.**
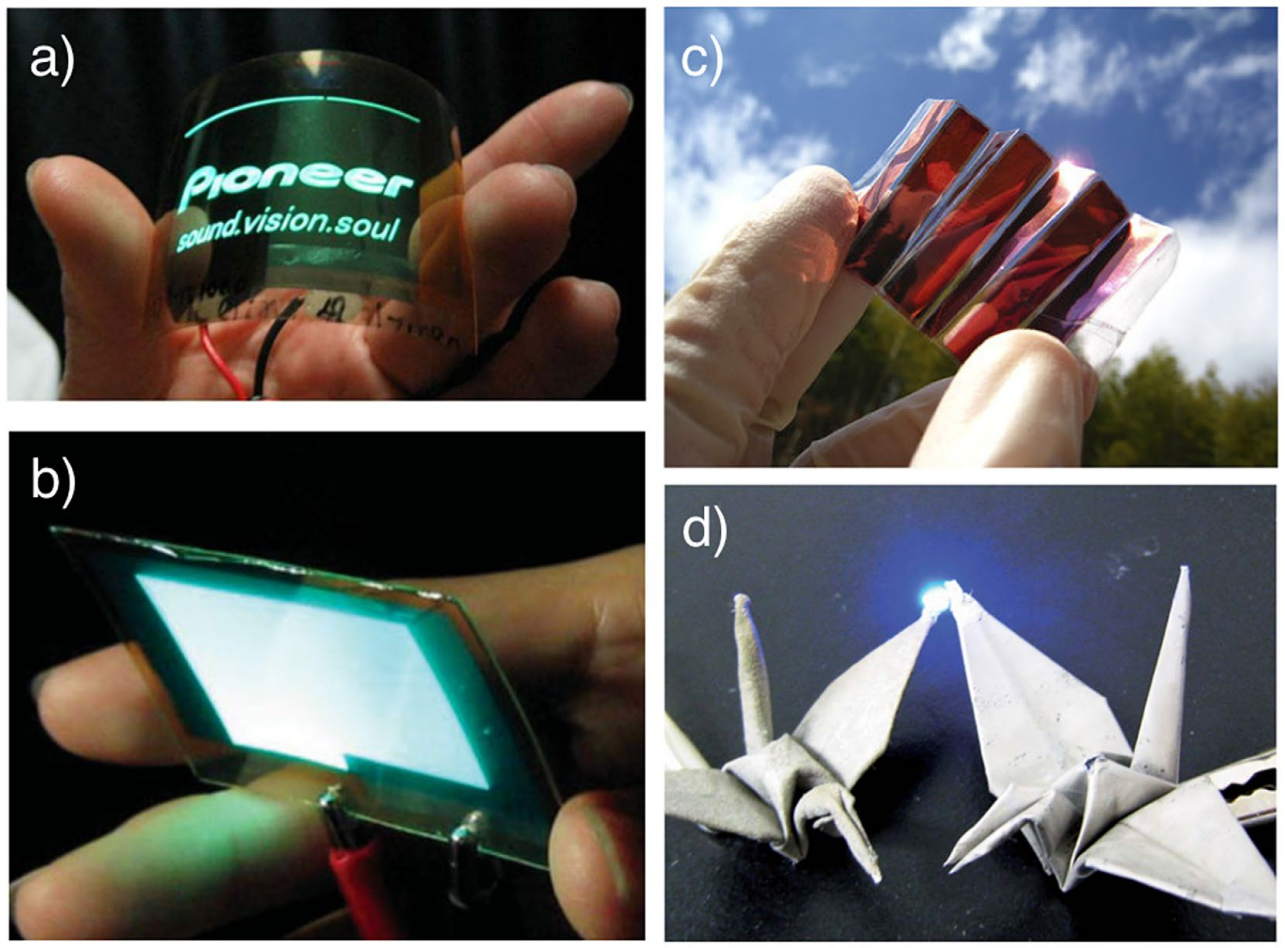
Application of NC as a base material for future flexible electronics. (a) A wooden NC composite film for the base of OLEDs. Reprinted with permission from [62]. Copyright 2009 Elsevier. (b) A BC nanocomposite for OLEDs. Reprinted with permission from [61]. Copyright 2008 John Wiley and Sons. (c) Conductive cellulose nanopaper for the base of foldable solar cells. Reprinted with permission from [67]. Copyright 2015 Nature Publishing Group. (d) Conductive nanopaper (left) and pulp paper (right) turning on an LED. Reprinted with permission from [69]. Copyright 2013 Royal Society of Chemistry.

Another application of NCs is the base material for future flexible electronics (Figure 3). NCs form flexible non-woven sheets called ‘nanopaper’ by traditional paper-making filtration treatment [7]. Some nanopapers are transparent with a very low CTE and high mechanical performance both before [58] and after mixing with resins [59–62]. Such flexible films are expected to be transparent substrates for future thin electronics (paper electronics) [63,64], including organic light-emitting diode (OLED) displays [62], flexible nonvolatile memory [65], transparent conductive films [66], foldable solar cells [67], and flexible antennae [68,69].

For paper electronics, exhaust heat management is required to cool the evolving elements and protect them from accidental thermal runaway or thermal fractures [70]. However, thinner devices have less volume for mounting general bulky heat sinks, so a different cooling mechanism is required to diffuse heat into the base substrate. In this mechanism, the substrate needs to spread heat in the in-plane direction, so the in-plane thermal conductivity becomes important [71]. An example of such a two-dimensional (2D) cooling material is the Japanese traditional craft *yuton*, a cooling floor mat used in summer and designated as an intangible folklore cultural asset by Fukui Prefecture. *Yuton* is made of the laminated Japanese traditional paper *washi* coated in perilla oil or lacquer [72]. Its cooling capacity is high and it has been said that one can catch a cold from inadvertently taking a nap on *yuton* [72]. The cooling mechanism of *yuton* is not clear, but one theory is that *yuton* absorbs sweat or moisture and smoothly vaporizes them to absorb the heat of vaporization and cool. Although the mechanism is not the same, the cooling substrates for paper electronics described above are the modern version of *yuton*, and it has been proposed that they can be fabricated using cellulose by investigating the heat-conducting properties of NCs [71,73–76].

In terms of heat transfer engineering, the state where the temperature *T* of homogeneous substances changes with time is called the non-equilibrium state, while the state where *T* remains constant is called the steady state. In the case of a one-dimensional steady temperature field, the heat flux *q* per unit time is proportional to the temperature gradient *∂T/∂x* in the *x*-direction, and the proportional constant is termed as the thermal conductivity *κ*. This corresponds to Fourier’s law, as described as:


(1)
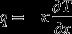



The *κ* therefore corresponds to the diffusion factor of the thermal energy within the substances. However, the heat-transferring phenomenon is also considered as the temperature diffusing process, and its diffusion coefficient is termed as the thermal diffusivity *α*. The temperature measurement is easy and most of the non-equilibrium methods are based on measuring *α.* The *α* is converted to *κ* by using the specific heat at a constant pressure *C*_p_ and density *ρ* as follows:


(2)
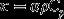



In this review, we first describe methods for measuring the thermal conductivity of 2D planar materials. We then give an overview of the recent reports of the heat-conducting properties of polymers and composites to give a better introduction to NC materials.

## 2. Thermal conductivity measurement methods for planar materials

Methods to measure the *κ* or *α* fall into the general classification of steady-state and non-equilibrium methods. Heating methods, including Joule heating and optical heating, are also used for further categorization. Non-equilibrium methods are classified by the measuring time scale (e.g. pulse heating, periodic heating, or step heating) and the signal detection procedure, including radiation thermometry, thermocouples, resistance thermometry, and the photoacoustic method.

The measurement system significantly restricts the specimen morphology (e.g. fibrous, planar, and bulky shapes) and the heat flux direction (i.e. the measuring direction of the sample). In particular, for anisotropic 2D planar samples, such as papers, sheets, and films, it is necessary to independently measure the in-plane and through-plane directions, although anisotropic (particularly in-plane) measurements are still unstandardized. Table 1 lists the reported measurement techniques for 2D materials.

**Table 1.** Thermal conductivity measurement techniques for 2D planar film materials.

Method	Thermal conductivity/diffusivity	Measuring direction	Measuring materials	References
Steady-state method	*κ*	In-plane	Nanofibrillated cellulose (NFC) and boron nitride composites	[77]
Steady-state bridge method	*κ*	In-plane	Cellulose nanocrystal (CNC) films	[71]
Laser spot periodic heating radiation thermometry method	*α*	In-plane and through-plane	NC sheets	[73,75]
			NC and resin composites	[74]
			Rubber and carbon fibre composites	[78]
	
Laser flash method	*α*	Through-plane	Rubber and carbon fibre compositesCNF and boron nitride nanotube composites	[78][76]
		In-plane		
Thermal wave analysis	*α*	Through-plane	CNF and resin composites	[56]
Alternating current (AC) calorimetric method	*α*	In-plane	CNF and resin composites	[56]
Temperature wave analysis with AC Joule heating method	*α*	Through-plane	Polymer films	[79–81]
			Papers	[82]

### 2.1. Steady-state methods

Steady-state methods apply a temperature gradient to the test sample and directly measure the thermal conductivity from the heat flux density. Some studies have uniquely applied steady-state measurements corresponding to the sample dimensions or measuring directions [71,77]. Steady-state methods have some drawbacks, such as heat leakage to the surroundings because of applying steady heat flow and relatively long measurement time because you need to wait until the temperature gradient of the sample reaches the steady state. In addition, the temperature difference needs to be detected with high accuracy, so it is difficult to measure thin or high-*κ* samples that give small temperature differences.

### 2.2. Laser spot periodic heating radiation thermometry method

In contrast to steady-state methods, non-equilibrium methods produce a temperature change on the sample surface and detect the temperature response on the back side of the sample [83]. This type of measurement allows measurement of the thermal diffusivity of relatively small samples in a short time. Even samples with high thermal diffusivities are measurable. However, the specific heat capacity and density of the sample are also needed to calculate the thermal conductivity.

**Figure 4.**
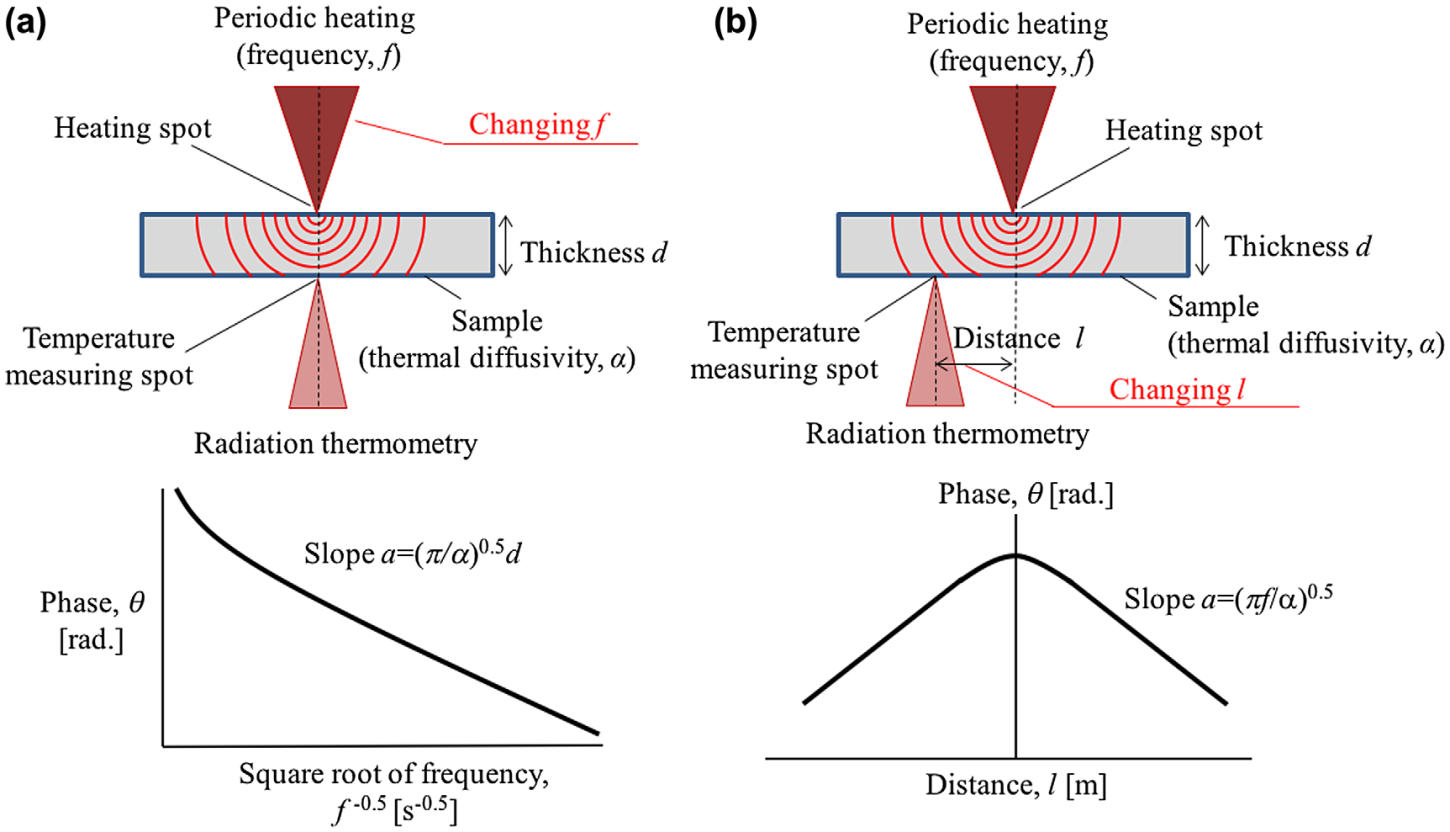
Measurement setup of the laser spot periodic heating radiation thermometry method. (a) The thermal diffusivity in the through-plane direction for 2D planar samples can be determined by the temperature response at the direct back side of the heating point with changing heating frequency *f*. (b) The thermal diffusivity in the in-plane direction needs to be detected at a distance *l* from the heating spot.

The laser spot periodic heating radiation thermometry method is a contactless method that independently measures both the in-plane and through-plane thermal diffusivities of 2D planar samples [73–75,78]. The surface of the sample is heated by an intensity-modulated laser beam and a radiation thermometer detects the back side temperature response, as shown in Figure 4. The heating frequency is changed to measure *α* in the through-plane direction, whereas the distance from the back side of the heating spot to the measuring spot is changed to measure *α* in the in-plane direction.

The sample requires thin blackening or metal-sputtering treatment to absorb the heating laser on the surface and avoid laser penetration or distance errors, and also to maintain high emissivity for the radiation thermometer to detect a sufficient signal on the back side. For a sample with a high *α*, such as a metal, the thickness of the blackening layer should be sufficiently small compared with the sample thickness because it sensitively affects *α* measurements and easily decreases the *α* values. In contrast, a relatively thick blackening layer has an insignificant effect on measuring *α* for small *α* samples, such as polymers. There is also large flexibility in the sample morphology because of the spotting periodic heating method. By adequately controlling the heating frequency so the temperature wave does not reach the sample edges, boundary conditions do not need to be considered.

### 2.3. Flash method

The flash method measures the thermal diffusivity by the half-time method [84]. In the flash method, one-dimensional thermal flow needs to be produced by heating the entire surface of the solid sample with pulsed light. The pulse width needs to be negligibly small compared with the half-time. The edges of the sample need to be held to avoid heat leakage. The flash method is applicable to solid samples that are assumed to be uniform structures, and the thermal diffusivity needs to be measured in the through-plane direction of the sample. A specialized attachment is required to measure the thermal diffusivity in the in-plane direction of the sample. Blackening treatment of the sample is also required, as is the case with the laser spot periodic heating radiation thermometry method.

## 3. Thermal conductivity of polymeric materials

Here, the thermal conductivity characteristics of general polymers and composites are briefly reviewed for the introduction and comparison of cellulose and NC materials.

### 3.1. Polymers

**Figure 5.**
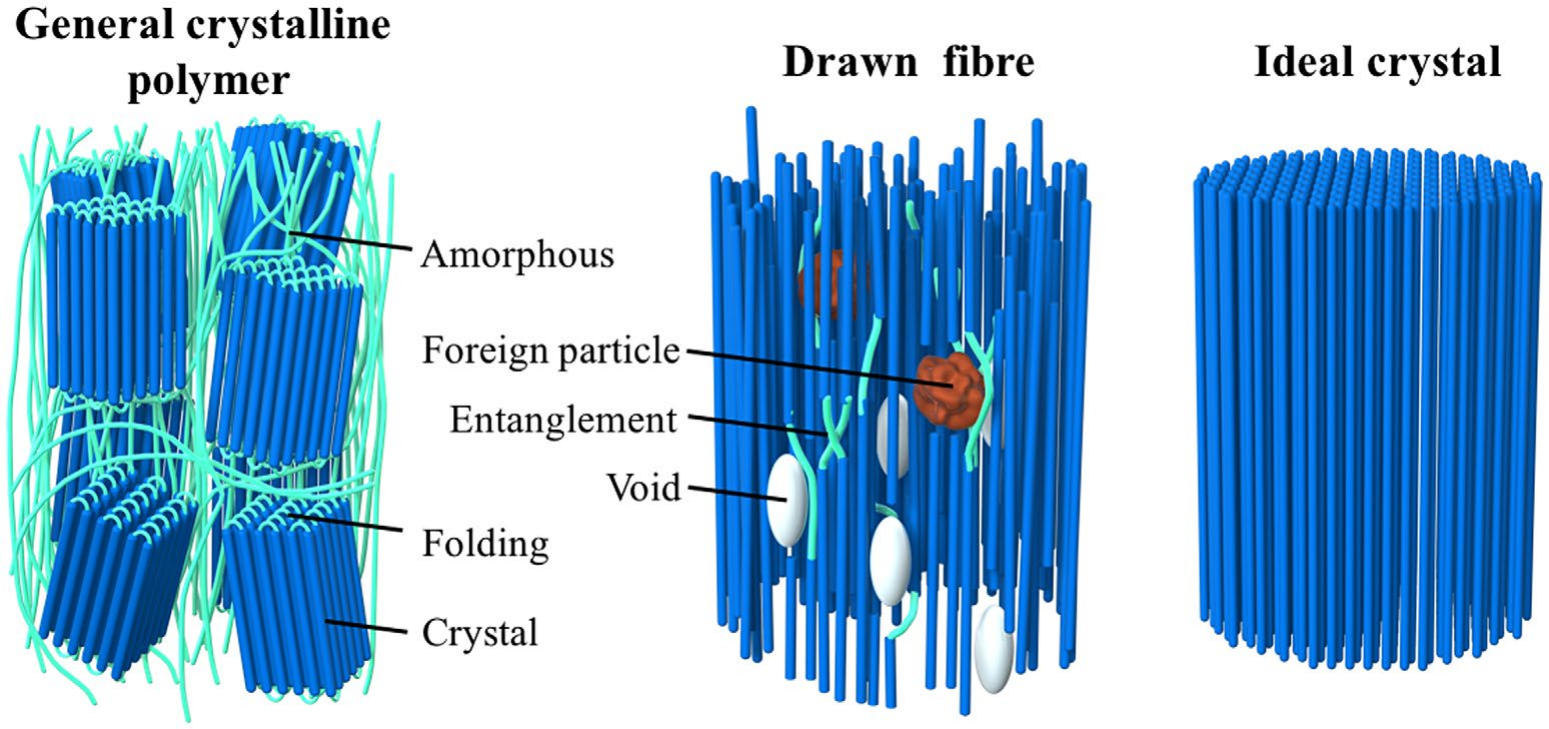
Schematic diagrams of the molecular structures of a general crystalline polymer, a drawn fibre, and an ideal polymer crystal.

General bulk polymers are known to have low *κ* values of 0.1–0.5 W/mK [85] because they contain defective structures, such as amorphous regions, voids, chain ends, and entanglements [86], which inhibit heat propagation (see Figure 5). The weak interaction forces, mainly van der Waals forces, and randomly oriented chain structures make the average phonon mean free path and the resulting thermal conductivity smaller than those of well-ordered polymer crystals. The chain stiffness, morphology [87,88], and phase transition behaviour [89] also affect the thermal conductivity of polymers. Tian et al. [90] reported that regenerated cellulose fibres have *κ* = 0.062 W/mK with *α* = 0.132 mm^2^/s[Bibr CIT0090].

The polymer chain dynamics also affect temperature propagation. The thermal diffusivity of amorphous polymers shows temperature dependence and a discontinuous change at the glass transition [79,91,92]. The change of the thermal diffusivity during the glass transition also increases as the glass transition temperature increases [79].

When the polymer chains align, the thermal conductivity in the chain axis direction is greatly enhanced by the formation of highly crystalline structures. It has been found that the thermal conductivity of polyethylene fibres increases as the tensile modulus increases, which is related to the thermal conductivity of the continuous crystal regions [93]. Shen et al. [94] reported that the thermal conductivity of polyethylene (bulk *κ* = ~0.35 W/mK) becomes very high after extreme drawing (*κ* = ~104 W/mK) and the nanofibres have a structure that is close to the ‘ideal’ single crystalline structure[Bibr CIT0094]. Interestingly, even when they are not highly crystalline, chain-oriented amorphous polythiophene nanofibres have thermal conductivity as high as ~4.4 W/mK owing to their short-range order [95]. Stretched spider silk has thermal conductivity as high as 416 W/mK by rearrangement of the intrachain and interchain hydrogen bonding to align the crystalline *β*-pleated sheets of the peptide chains [96].

**Figure 6.**
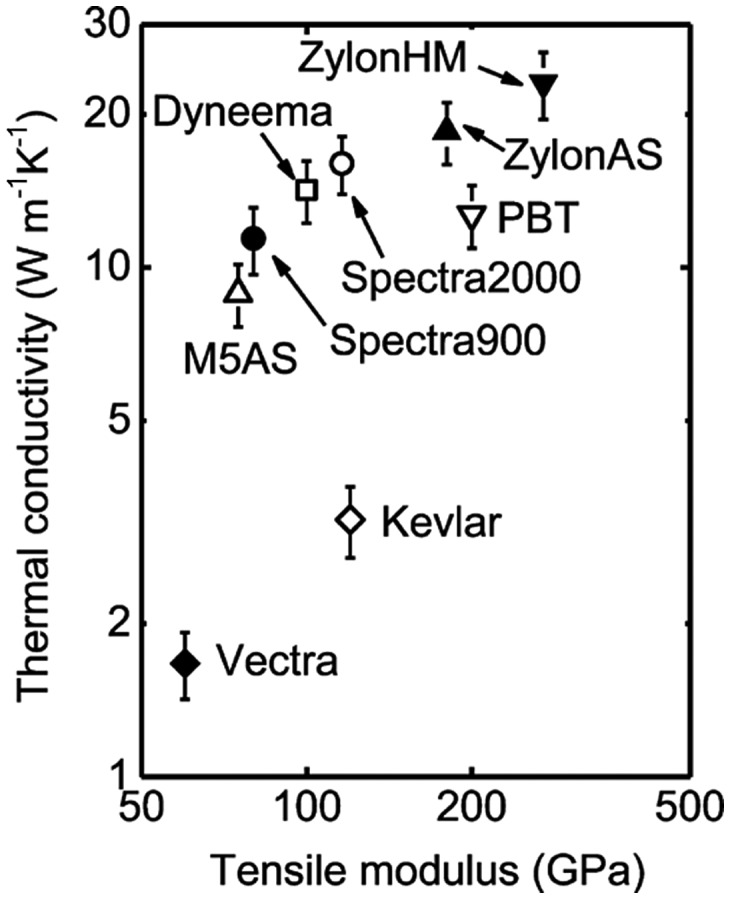
Relationship between the thermal conductivity and tensile modulus for various polymer fibres. Reprinted with permission from [98]. Copyright 2013 American Chemical Society.

The relationship between the Young’s modulus and the thermal conductivity of drawn polymers has been investigated. Choy et al. [97] found that the axial Young’s modulus of drawn polyethylene increases with increasing thermal conductivity[Bibr CIT0097]. Wang et al. [98] found that the thermal conductivities of various crystalline polymer fibres (known as high-modulus fibres) are roughly correlated with the tensile modulus[Bibr CIT0098], as shown in Figure 6. Although the authors suggested that the thermal conductivity is not only directly controlled by the tensile modulus [98], ‘bulk’ cellulose I crystals may have relatively high thermal conductivity when considering their high modulus of 140–150 GPa [33,34].

### 3.2. Composites

To develop thermal management materials such as thermal interface materials (TIMs), which physically connect heat sources with heat sinks, the introduction of various inorganic, metallic, ceramic, and carbonaceous [99] fillers can enhance the thermal conductivity of polymers [85]. The thermal conductivity of composites increases with increasing filler loading (e.g. [100–102]). The thermal conductivity enhancement (TCE, %) is used to evaluate the filler effect at a specific loading fraction. For example, Shahil and Balandin [103] reported that epoxy composites with a graphene and multilayer graphene hybrid filler have a large TCE of 2300% at a small filler loading of 10%, which gives *κ* ≈ 5.1 W/mK[Bibr CIT0103]. Introducing hybrid fillers with different sizes [104–107] or species [103,108,109] is effective for enhancing the thermal conductivity of composites.

Percolative filler networks are also effective for enhancing thermal conductivity [107,110]. However, high concentrations or fillers with rigid networks impair the softness of materials and hinder their use as TIMs, which need to adhere tightly to both the rigid heat source and heat sink, as well as reduce the thermal resistance with high through-plane thermal conductivity. Fluorinated rubber mixed with vertically aligned carbon fibres by electrostatic flocking exhibits through-plane thermal conductivity of ~23 W/mK at low filler loading of ~13 wt% with TCE of above 10,000% [78]. The addition of anisotropic filler structures also results in anisotropic TCE of composites [78,111–113]. Penetration of the filler structures into the TIM surface plays an important role in achieving high thermal conductivity [78,111,113].

Compressible TIMs have been reported to express higher through-plane thermal conductivity performance after deformation [114,115]. For example, graphene/carbon nanotube aerogel has *κ* = 88.5 W/mK after compression with thermal interface resistance of 13.6 mm^2^/KW at an initial density of 85 mg/cm^3^ [116].

Celluloses are used as matrices to produce thermally conductive composites with additional functions, including mechanical properties [90] and transparency. Zeng et al. [76] mixed boron nitride nanotubes (BNNT) with a CNF matrix to produce composite films, which enhanced the in-plane thermal conductivity to 21.39 W/mK at 25 wt% BNNT loading compared with that of the neat CNF sheet of 1.45 W/mK[Bibr CIT0076]. Zhu et al. [77] reported that the thermal conductivity of TEMPO-mediated wooden NFC sheets (*κ* = 0.035 W/mK) can be enhanced to 26.2 and 145.7 W/mK with boron nitride (BN) loadings of 5 and 50 wt%, respectively[Bibr CIT0077]. Similarly, Zhou et al. [117] coated a thin layer of BN nanosheets on wooden CNF nanopaper (in-plane *κ* = 0.04 W/mK) and achieved optical transparency of 70% and in-plane *κ* = 0.76 W/mK at a BN content of 2.5 wt%[Bibr CIT0117]. Such 2D heat-spreading materials show potential as the thermal dissipation substrates of electronics to protect from thermal failure [76,77].

NC has also been applied as a TCE filler for composites. Shimazaki et al. [56] reported that with a fibre content of 58 wt%, NFC sheet/epoxy resin composites show enhanced thermal conductivities of 0.23 and 1.1 W/mK in the through-plane and in-plane directions, respectively, compared with the neat epoxy resin (isotropic *κ* = 0.15 W/mK)[Bibr CIT0056]. By mixing 15 wt% CNWs with polypropylene (PP) and a maleic anhydride grafted PP mixture matrix, the thermal conductivity of PP (*κ* = 0.2675 W/mK) can be increased to 0.4194 W/mK [57].

### 3.3. Paper

Morikawa and Hashimoto [82] reported through-plane thermal diffusivity measurements of typical cellulosic paper coated with kaolinite by applying the AC Joule heating method[Bibr CIT0082]. The thermal diffusivity of the paper linearly decreased as the bulk density increased at 50 °C, resulting in the thermal conductivity remaining almost constant (*κ* ≈ 0.105 W/mK) for different bulk densities. They concluded that the air within the pulp networks effectively transports the temperature wave rather than the thermal energy [82], because air has a higher thermal diffusivity than polymers.

## 4. Thermal conductivity of NC materials

Four structural factors are considered to affect the heat-conducting behaviour of cellulose nanopaper: crystallite thickness; fibre orientation; interfacial thermal resistance between fibres; and small pores.

### 4.1. Crystallite size effect

**Figure 7.**
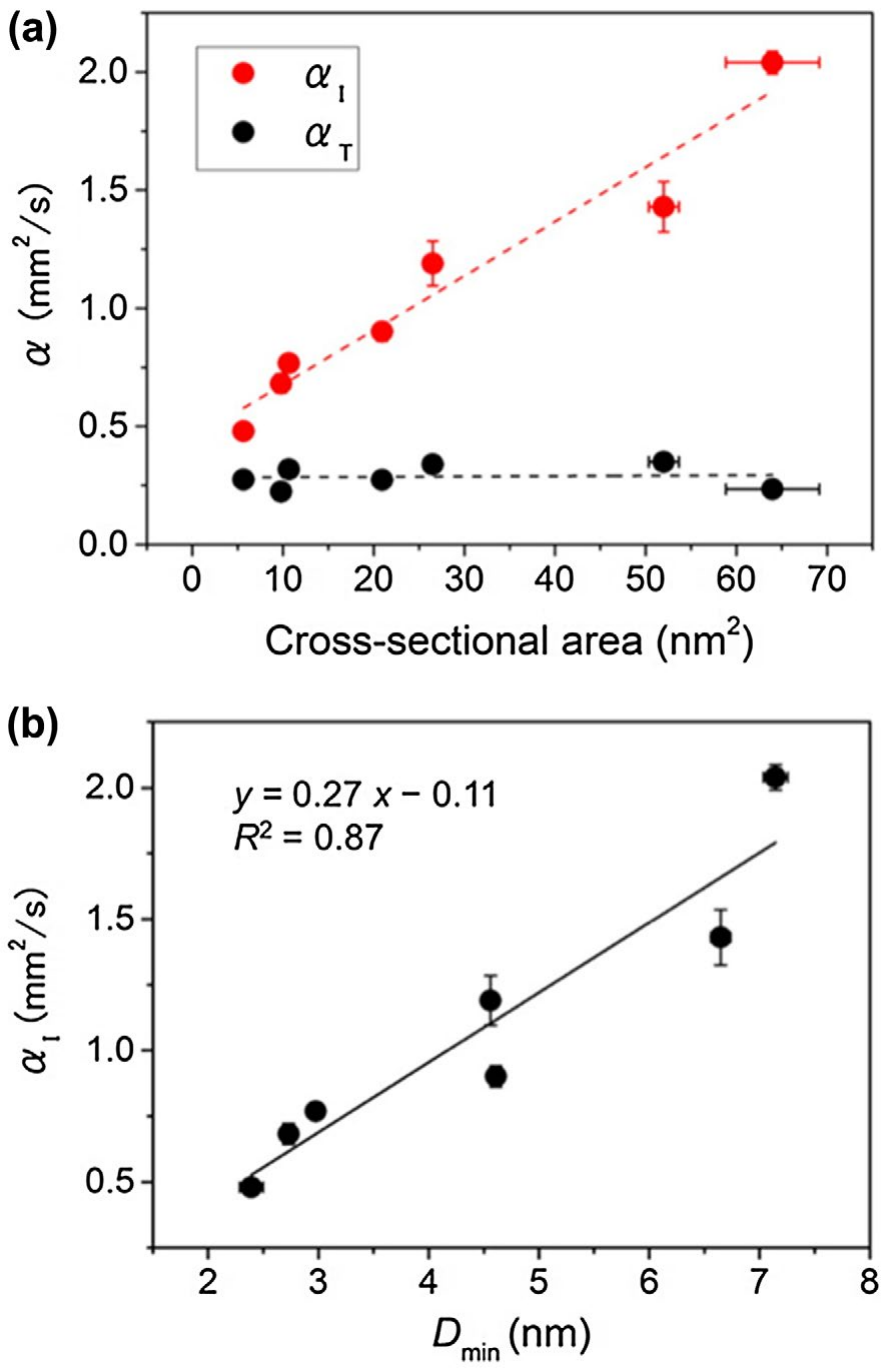
Crystallite size effect for cellulose nanopaper. (a) Relationship between the cross-sectional area of crystalline cellulose obtained by X-ray diffractometry (XRD) and the thermal diffusivity of nanopapers in the in-plane (*α*_I_) and through-plane (*α*_T_) directions. (b) Relationship between the minimum crystallite width *D*_min_ determined by XRD and *α*_I_. Reprinted with permission from [73]. Copyright 2015 American Chemical Society.

Uetani et al. [73] reported the heat-conducting properties (*κ* and *α*) and their anisotropy in the in-plane and through-plane directions of non-woven NC sheets (cellulose nanopaper) made of various materials, including tunicate, BC, cotton, and wood pulp (see Figure 1)[Bibr CIT0073]. The through-plane thermal conductivities of these nanopapers are similar (0.3–0.5 W/mK) despite the different NC species, whereas the in-plane thermal conductivities are different (0.6–2.5 W/mK). The authors found that the crystallite sizes of the NCs, including the width and the cross-sectional area, estimated by wide-angle X-ray scattering are well correlated with the in-plane thermal diffusivity [73], as shown in Figure 7. On the other hand, the relative degree of crystallinity was found to have an indirect effect on the in-plane thermal diffusivity. The nanopaper with thicker fibres (larger crystallite size) shows higher in-plane thermal diffusivity: this relationship is called the crystallite size effect.

**Figure 8.**
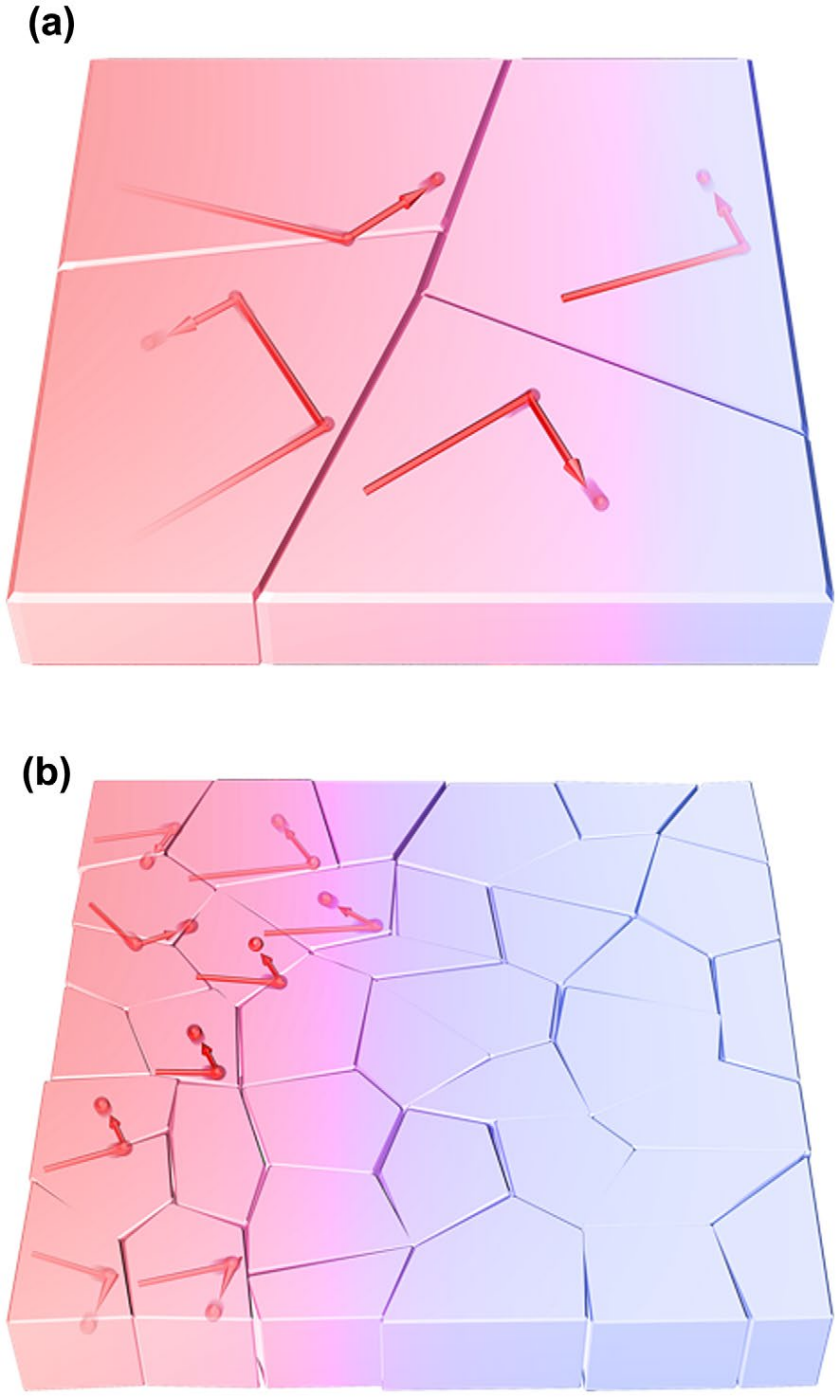
Schematic diagram of the size effect for phonon transfer within crystalline grains. (a) The phonon is diffusively transported within large grains compared with the phonon mean free path. (b) Ballistic transportation becomes dominant within small grains.

**Figure 9.**
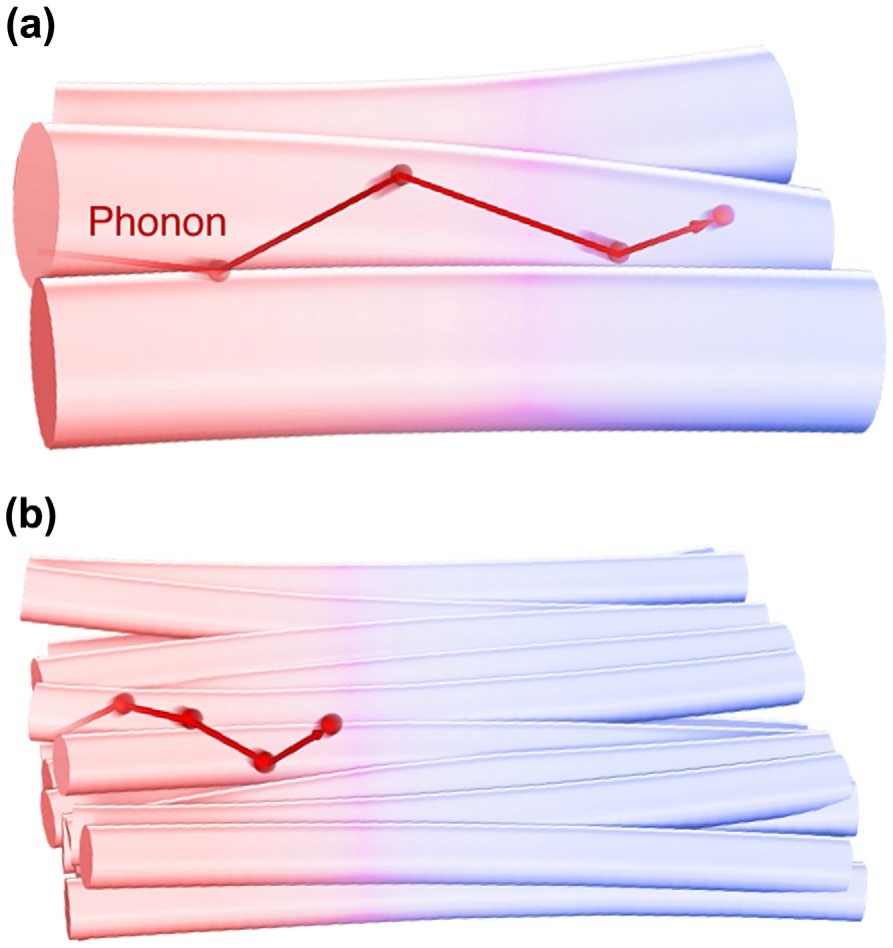
Schematic diagram of the size effect for phonon transfer within crystalline fibres. The phonon mean free path is longer within the thicker fibre aggregates (a) than within the thinner fibre aggregates (b), and the resulting thermal conductivity is directly affected.

The size effect is a known phenomenon when considering the thermophysical properties at the micro/nanoscale [118]. We will now discuss thermal conductivity reduction because of nanoscale structures. In terms of the phonon gas kinetic model, the thermal conductivity can be described by(3)
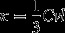



where *C*, *v*, and *l* are the specific heat capacity, phonon group velocity, and phonon mean free path, respectively. As shown in Figure 8(a), when the grain size *L* (i.e. the representative size of the system) is sufficiently larger than *l* (Knudsen number *Kn* = *l*/*L* ≪ 1), phonons would be scattered by other phonons (Umklapp scattering) before reaching the grain boundary, resulting in that grain having the same thermal conductivity as the bulk single crystal. This is called diffusive phonon transportation, where the thermal conductivity is determined by the specific heat capacity or the phonon group velocity. Conversely, when the grain size is smaller than *l* (Figure 8(b)), phonons are scattered by the grain boundary rather than the other phonons (ballistic phonon transport) and *l* decreases owing to structural limitation, resulting in the thermal conductivity of the grain being smaller than that of the bulk single crystal. However, it should be noted that the phonon mean free path is affected by the phonon frequency, wave number, and polarization. Therefore, the actual transportation mode is considered to be quasi-ballistic, where diffusive and ballistic modes coexist. The simple concept of the size effect is applicable to crystalline nanofibrous aggregates (nanopaper) [73] in addition to the thin materials of polymers [119,120], metals [121–123], and carbon materials [124,125], which is schematically illustrated in Figure 9. The NC fibre thickness corresponding to the crystallite size limits *l* to reduce the thermal conductivities of single fibres and the sheet.

### 4.2. NC fibre orientations

**Figure 10.**
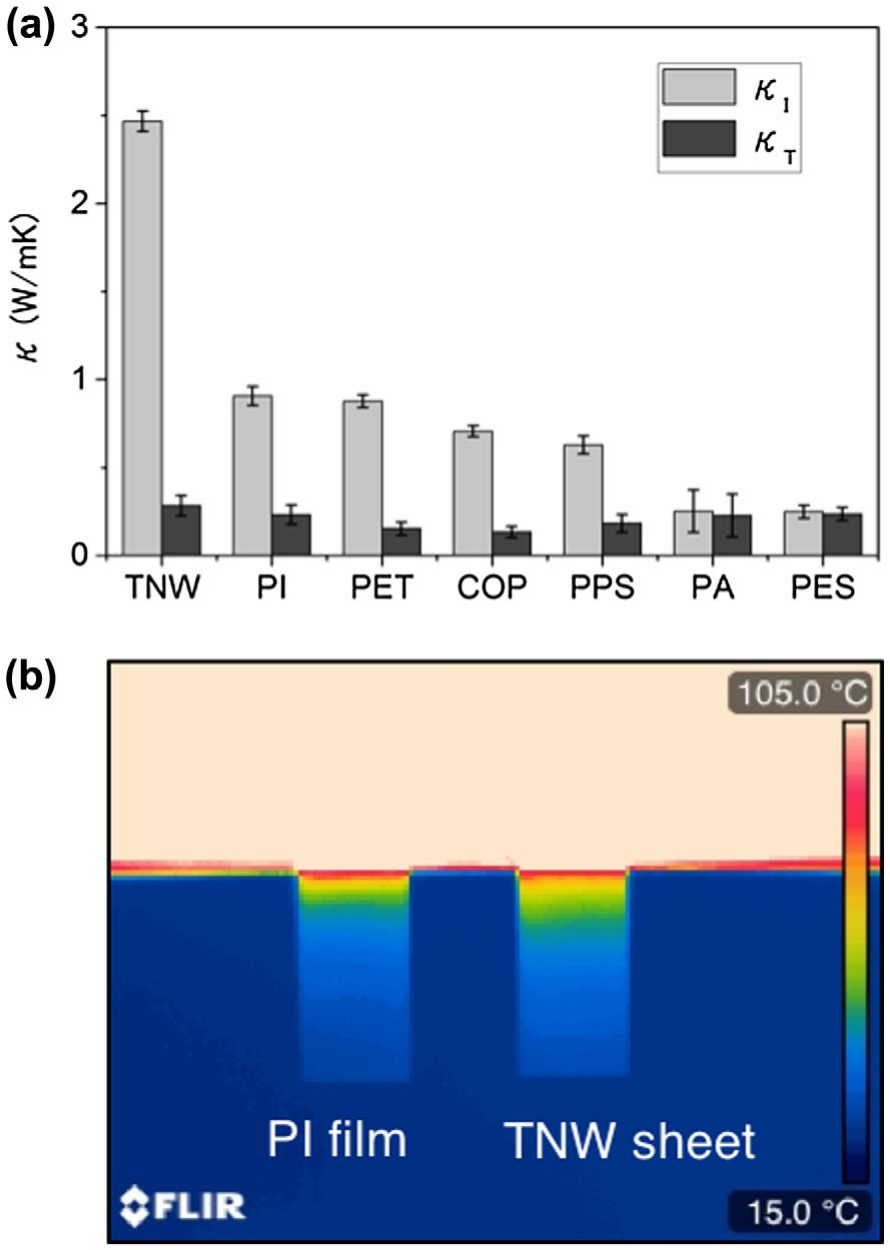
Comparison of the thermal conductivity of cellulose nanopaper and plastic films. (a) Nanopaper derived from TNWs shows 3–10 times higher in-plane thermal conductivity (*κ*_I_) of 2.5 W/mK. Abbreviations: PET – polyethylene terephthalate, COP – cyclo-olefin polymer, PPS – polyphenylene sulfide, PS – polyamide, and PES – poly(ether sulfone). (b) Thermograph of a polyimide (PI) film and a TNW sheet (nanopaper). Cellulose transfers the heat for a longer distance than the PI film. Reprinted with permission from [73]. Copyright 2015 American Chemical Society.

Nanopaper made of thick tunicate nanowhiskers (TNWs) exhibits in-plane thermal conductivity of 2.5 W/mK, which is 3–10 times higher than that of conventional plastic films (Figure 10(a)) [73]. In addition, the through-plane thermal conductivity of the same nanopapers is as low as ~0.3 W/mK. This large thermal conductivity anisotropy is considered to be because of the NC fibre orientation.

**Figure 11.**
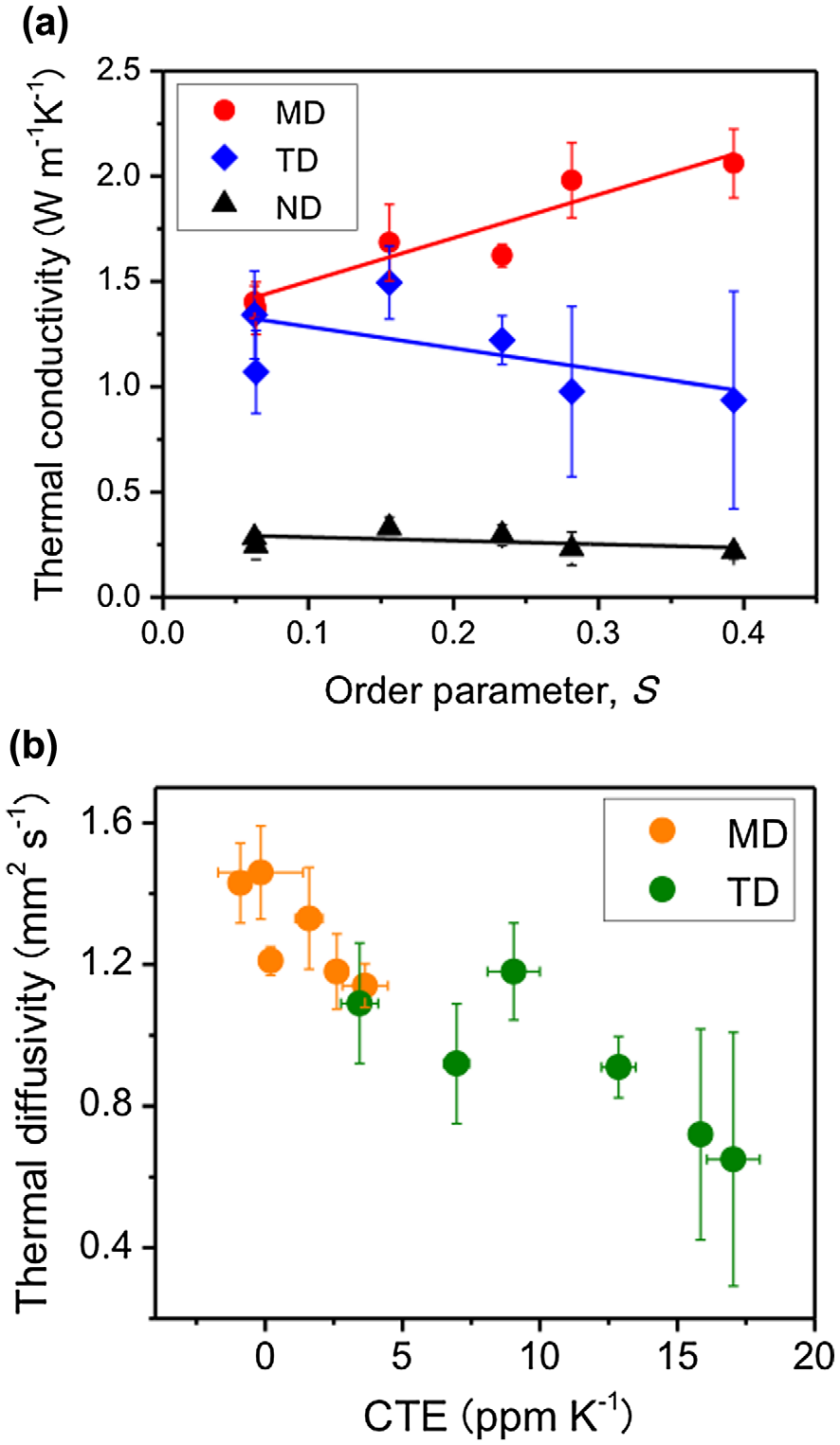
Effect of the fibre orientation on the thermal properties. (a) Relationship between the orientational order parameter *S* of stretched BC nanopapers and the thermal conductivity in the MD (stretching direction), TD, and ND (through-plane direction). (b) Relationship between the CTE and the thermal diffusivity. The CTE is inversely correlated with the thermal diffusivity regardless of the nanopaper direction. Reprinted with permission from [75]. Copyright 2017 American Chemical Society.

Uetani et al. [75] attempted to align the NC fibres within the nanopaper planes by stretching BC hydrogels (known as *nata de coco*)[Bibr CIT0075]. As the orientational order parameter *S* of the nanopaper determined by X-ray diffraction increased, the *κ* value in the stretched direction (machine direction, MD) increased and that in the transverse direction (TD) decreased (Figure 11(a)). These results show that the NC fibres have thermal conductivity anisotropy between the longitudinal and thickness directions.

Considering that *κ* is proportional to *S*, the authors estimated that the thermal conductivity anisotropies of both the ideal BC nanopapers and single BC nanofibres are about 10 times by roughly extrapolating to the hypothetical *κ* value at *S* = 1 where NC fibres perfectly align. The thermal conductivity anisotropy of ideal sheets with perfect NC alignment is thought to reflect that of single NC fibres, because the heat irradiated on the sheet surface and detected on the back side is thought to pass the same number of interfaces of NC fibres in alignment direction (MD) and through-plane direction (normal direction, ND).

They also found that there is an inverse relationship between the thermal diffusivity and the CTE (Figure 11(b)) [75]. They concluded that heat conduction is sensitively affected by the CTE and phonons can be scattered by the dimensional instability within the nanopaper.

### 4.3. Interfacial thermal resistance between NC fibres

**Figure 12.**
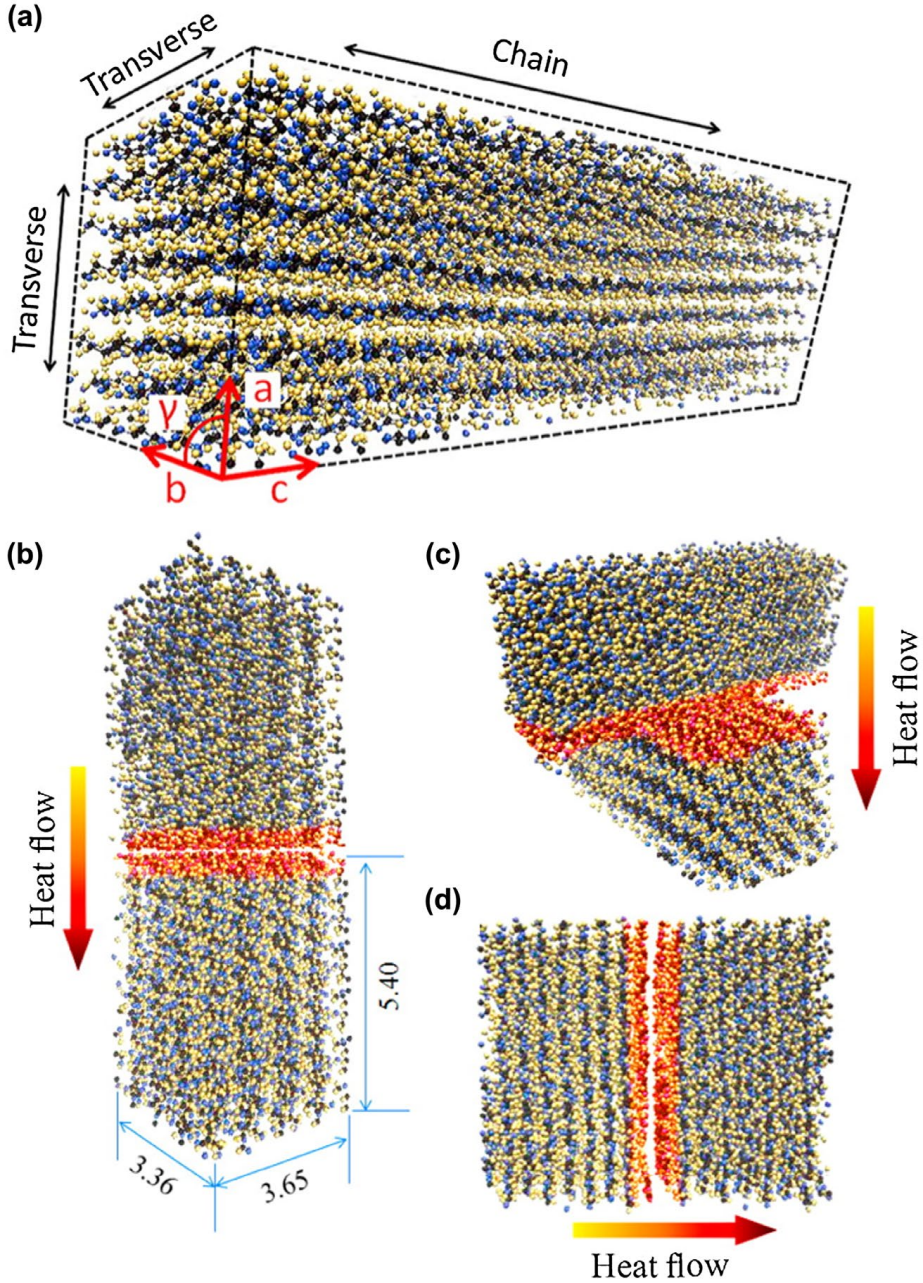
(a) Cellulose I*β* crystal structure used to analyse the interfacial thermal resistance in molecular dynamics simulations with (b)–(d) various contact positions. Distances are in nm. Reprinted with permission from [71]. Copyright 2014 American Chemical Society.

Nanopapers with NC fibre aggregates contain a lot of fibre interfaces, and the interfacial thermal resistance is not negligible. Because direct measurement of the interfacial thermal resistance is technically difficult, Diaz et al. [71] performed molecular dynamics simulations to investigate the interfacial thermal resistance of cellulose I*β* crystals. They calculated the thermal conductivity of a cellulose I*β* crystal (Figure 12(a)) using molecular dynamics simulations and predicted that the thermal conductivities in the chain and transverse directions are ~5.7 and ~0.72 W/mK, respectively. This ~10-fold difference in the conductivity is considered to be correlated with the intrinsic crystalline anisotropy. They also modelled the thermal conduction between two adjacent crystals, as shown in Figures 12(b)–(d), and calculated the range of the thermal resistance to be 9.4–12.5 m^2^ K/GW [71]. These values are smaller than the Kapitza resistance of the thermoelectric Si_0.7_Ge_0.3_ alloy (~95 m^2^ K/GW) [126] and single-walled carbon nanotubes (70–110 m^2^ K/GW) [127]. They suggested that the lower interfacial thermal resistance between cellulose crystals is derived from the strong surface interactions, such as hydrogen bonding and van der Waals forces.

### 4.4. Small pores within nanopapers

Cellulose nanopapers contain many pores that trap air. They have a low thermal conductivity of ~0.026 W/mK and the mean free path of gas molecules is about 70 nm at ordinary temperature and pressure [47]. Considering the fibre widths or densities, the pores within nanopapers of even thick tunicate NC are presumed to be smaller than 70 nm based on theoretical prediction [128]. Uetani et al. [73] suggested that gas air molecules are unable to undergo convection heat transfer within nanopapers and the small pores are adiabatic[Bibr CIT0073].

### 4.5. Temperature dependence

**Figure 13.**
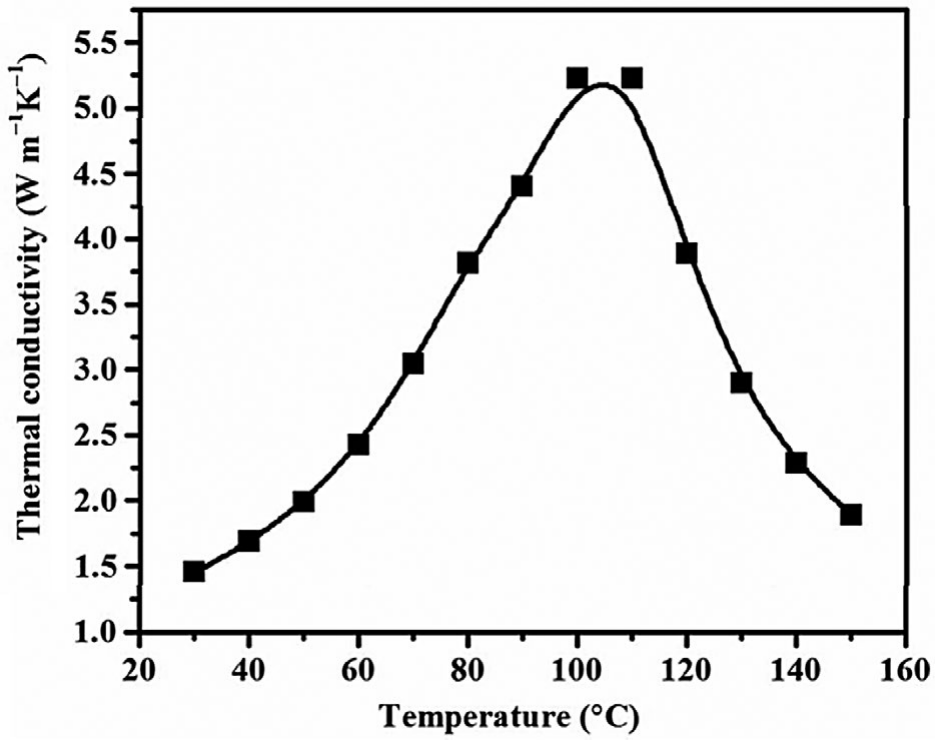
Relationship between the in-plane thermal conductivity and the temperature for pure CNFs. Reprinted with permission from [76]. Copyright 2017 American Chemical Society.

It has been reported that the temperature also affects the thermal conductivity of cellulose nanopapers. Zeng et al. [76] found that the in-plane thermal conductivity of pure CNFs at room temperature (~30 °C, *κ* ≈ 1.45 W/mK) drastically increases with increasing temperature until ~100 °C and it then decreases, as shown in Figure 13 [Bibr CIT0076]. They considered that the increase in the thermal conductivity at low temperature originates from the amorphous parts and the decrease at high temperature is because of Umklapp phonon scattering.

### 4.6. Comparison of the thermal conductivity and CTE of cellulose nanopapers with other materials

**Figure 14.**
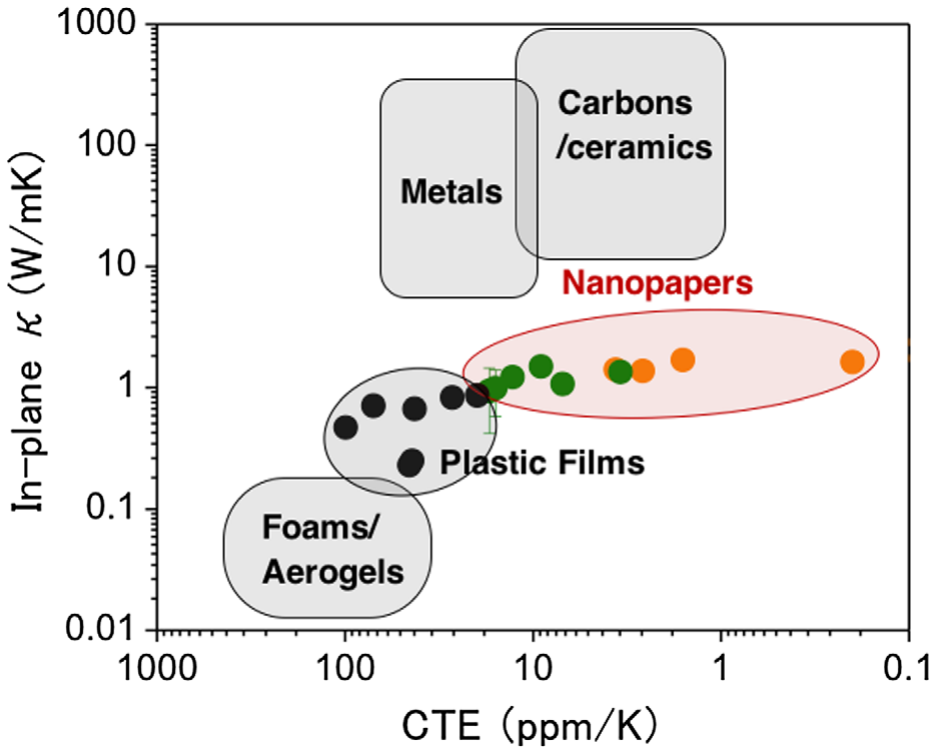
Ashby plot of CTE versus the in-plane thermal conductivity for various materials, including cellulose nanopapers [71]. The data for the plastic films and nanopapers were obtained from [73] and [75], respectively.

The Ashby plot in Figure 14 shows the positioning of the thermal conductivity and CTE of cellulose nanopapers among various materials. Nanopapers have higher *κ* and lower CTE values than plastic films [73,75]. Porous foams/aerogels have smaller CTE and *κ* values, whereas metals, carbon materials, and ceramics have much higher *κ* values and smaller CTE values [71]. Cellulose is therefore located in the middle of the thermal conductivity range with relatively small CTE. This region is occupied by polymer composites mixed with heat-conducting fillers, such as carbon materials or ceramics, which directly compete with cellulose nanopapers. To differentiate from their competitors, application development of cellulose nanopapers by exploiting the intrinsic features of NC is important.

## 5. Applications of thermally conductive NC films

### 5.1. Transparent materials

It is expected that thermally conductive cellulose nanopapers can be used as heat exhaust base materials for flexible electronics, such as printed circuit boards and light-emitting diodes. For such applications, films with both transparency and high thermal conductivity are required. Cellulose nanopapers are transparent both on their own [58] and when mixed with resins [61].

**Figure 15.**
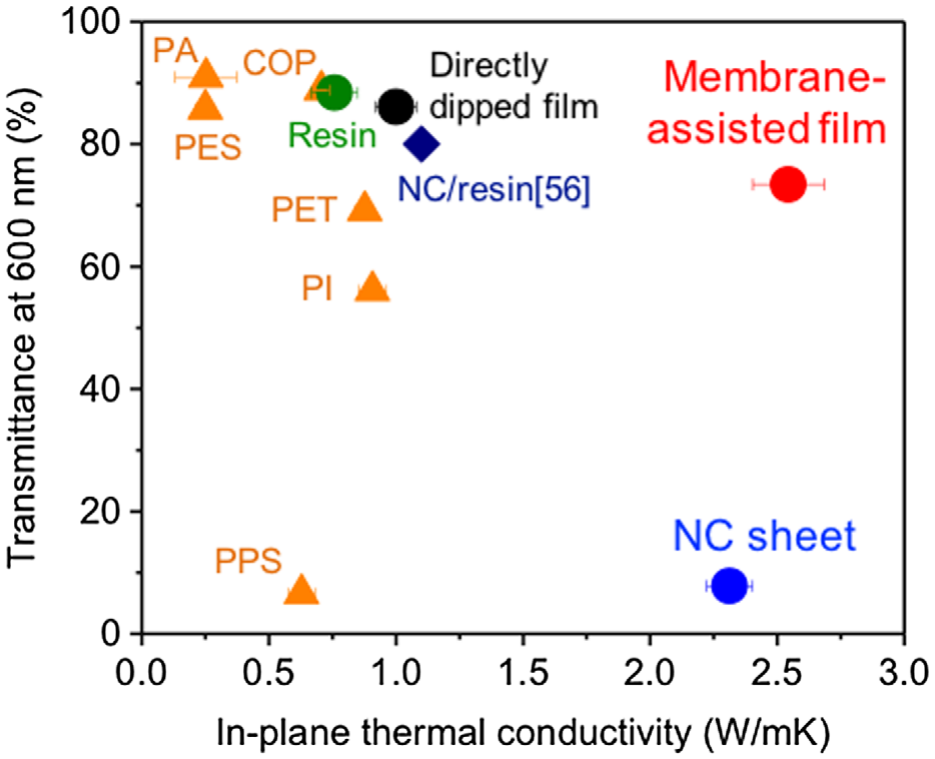
Relationship between the in-plane thermal conductivity and the transparency for plastic films and nanopaper materials [74]. Reproduced with permission from The Royal Society of Chemistry.

Uetani et al. [74] reported thermally conductive transparent NC films prepared by mixing transparent acrylic resin with the membrane-assisted method, which does not allow the formation of resin layers on the film surfaces[Bibr CIT0074]. The membrane-assisted film without resin layers on the film surfaces simultaneously has a high transparency of ~70% and in-plane thermal conductivity of ~2.5 W/mK by exploiting the intrinsic thermal conductivity of the skeletal NC sheet (Figure 15). This procedure allows the fibre content to be changed to control the resulting in-plane thermal conductivity and transparency.

### 5.2. Heat-guiding materials

**Figure 16.**
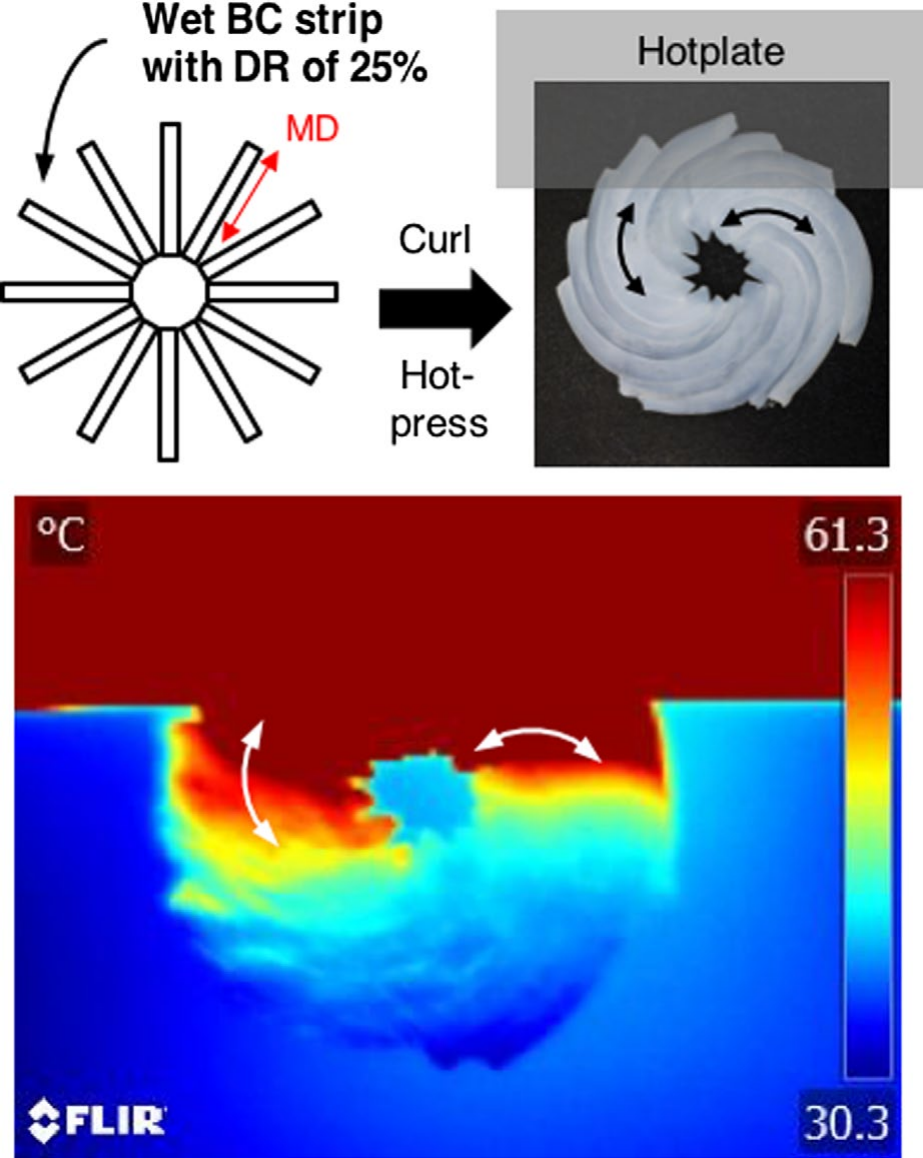
Heat-guiding materials produced by assembling stretched BC hydrogels with a draw ratio (DR) of 25% to form a planar spiral paper. Reprinted with permission from [75]. Copyright 2017 American Chemical Society.

To express the thermal conductivity anisotropy of NC fibres in macroscopic materials, Uetani et al. [75] created a planar spiral-shaped sheet by manually assembling drawn BC hydrogel stripes (draw ratio 25%, order parameter *S* ≈ 0.4), as shown in Figure 16 [Bibr CIT0075]. When this spiral paper was cantilevered between hot plates, an asymmetrical temperature distribution was revealed by infrared thermography. Heat circularly transferred along the fibre-aligning directions (left side of the spiral sheet, indicated by the double-headed arrows in Figure 16), while the drawn stripes in the transverse direction hardly conducted heat (right side of the sheet). They believe that heat-guiding materials can be produced by controlling the NC fibre alignment using the intrinsic thermal conductivity anisotropy.

## 6. Conclusions and outlook

Cellulose, which is currently used as a thermal insulator, exhibits better heat-conducting properties when transformed to NCs. This is the first example of bio-based thermally conductive materials that could contribute to the future development of sustainable heat-transfer structures. Living organisms that synthesize cellulose have thermal conductive elements in their tissues. Thus, from their anatomy it may be possible to learn about the hidden thermal management mechanisms. Future electronics could be effectively cooled by cellulose nanopapers by exploiting the potential of the intrinsic heat-conducting properties of NCs.

## Disclosure statement

No potential conflict of interest was reported by the authors.
